# Global review of the H5N8 avian influenza virus subtype

**DOI:** 10.3389/fmicb.2023.1200681

**Published:** 2023-06-02

**Authors:** Saba Rafique, Farooq Rashid, Sajda Mushtaq, Akbar Ali, Meng Li, Sisi Luo, Liji Xie, Zhixun Xie

**Affiliations:** ^1^SB Diagnostic Laboratory, Sadiq Poultry Pvt. Ltd, Rawalpindi, Pakistan; ^2^Department of Infectious Diseases, Chongqing Public Health Medical Center, Chongqing, China; ^3^Poultry Research Institute, Rawalpindi, Pakistan; ^4^Department of Biotechnology, Guangxi Veterinary Research Institute, Nanning, China; ^5^Guangxi Key Laboratory of Veterinary Biotechnology, Nanning, China; ^6^Key Laboratory of China (Guangxi)-ASEAN Cross-border Animal Disease Prevention and Control, Ministry of Agriculture and Rural Affairs of China, Nanning, China

**Keywords:** avian influenza virus, H5N8 subtype, epidemiology, surveillance, control and prevention

## Abstract

Orthomyxoviruses are negative-sense, RNA viruses with segmented genomes that are highly unstable due to reassortment. The highly pathogenic avian influenza (HPAI) subtype H5N8 emerged in wild birds in China. Since its emergence, it has posed a significant threat to poultry and human health. Poultry meat is considered an inexpensive source of protein, but due to outbreaks of HPAI H5N8 from migratory birds in commercial flocks, the poultry meat industry has been facing severe financial crises. This review focuses on occasional epidemics that have damaged food security and poultry production across Europe, Eurasia, the Middle East, Africa, and America. HPAI H5N8 viral sequences have been retrieved from GISAID and analyzed. Virulent HPAI H5N8 belongs to clade 2.3.4.4b, Gs/GD lineage, and has been a threat to the poultry industry and the public in several countries since its first introduction. Continent-wide outbreaks have revealed that this virus is spreading globally. Thus, continuous sero- and viro-surveillance both in commercial and wild birds, and strict biosecurity reduces the risk of the HPAI virus appearing. Furthermore, homologous vaccination practices in commercial poultry need to be introduced to overcome the introduction of emergent strains. This review clearly indicates that HPAI H5N8 is a continuous threat to poultry and people and that further regional epidemiological studies are needed.

## Introduction

Avian influenza viruses (AIVs) belong to the Orthomyxoviridae family and contain a segmented genome with eight single-stranded RNA segments and have negative polarity (Webster et al., 1992). Hemagglutinin (HA) gene and neuraminidase (NA) gene, two of the envelope proteins of these viruses, are used to classify them into different subtypes (Kawaoka et al., 1988). To date, 16 HA and 9 NA subtypes of AIVs have been identified in poultry and wild birds (Wang et al., 2022).

Low-pathogenic avian influenza (LPAI) viruses are naturally found in wild water birds such as swans, ducks, gulls, geese, swans, shorebirds, and terns (Krammer et al., 2018; Verhagen et al., 2021). LPAI viruses are transmitted to domestic birds, animals, and even humans from wild water birds. Influenza viruses with H5 HA have been circulating in wild birds and domestic poultry since 1995 (Harfoot and Webby, 2017). The Qinghai Lake-like H5N1 virus was first widely spread by migratory birds and caused huge damage to the poultry industry worldwide, but the origin of the virus remains unclear. The LPAI viruses of the H5 subtype, when infecting poultry, can evolve into HPAI viruses, causing severe mortality (Alexander and Brown, 2009). During July and August 2005, HPAI H5 clade 2.2 viruses were detected in poultry farms in Russia and Kazakhstan, where they caused high mortality (Coulombier et al., 2005). These viruses were genetically related to viruses detected in 2005 in Qinghai Lake in China (Chen et al., 2005). From July 2005 onward, HPAI H5 viruses were observed to cause outbreaks on poultry farms (Coulombier et al., 2005). The H5N1 virus became endemic in 2003 in Southern China, giving rise to several genotypes.

In mainland China, the H5N8 virus was detected in poultry between 2009 and 2010, which derived its HA gene from the Asian H5N1 lineage and its neuraminidase (NA), nucleoprotein (NP), and polymerase basic (PB1) genes from unidentified, non-H5N1 viruses. The H5N8 virus is highly pathogenic to chickens and moderately to extremely dangerous to mice (Zhao et al., 2013). In 2014, a novel reassortant HPAI H5N8 clade 2.3.3.4 virus with the HA gene was identified in South Korea (Jeong et al., 2014). Two types of H5N8 were found during these outbreaks, namely Gochang-like and Buan2-like. The predominant group, Buan2-like, afterward spread to Europe, East Asia, and North America by migratory waterfowl and formed three distinct subgroups (Jeong et al., 2014; Lee et al., 2014; Dalby and Iqbal, 2015; Lee et al., 2015; Verhagen et al., 2021). In autumn 2016, another High pathogenic AI H5N8 virus of clade 2.3.4.4 spread across different continents (Li et al., 2017) and showed sustained prevalence in Africa, Europe, and the Middle East (OIE, https://www.oie.int/en/animal-health-in-the-world/). In early 2020, HAPI H5N8 was continuously reported in Iraq, Kazakhstan, and Russia (Lewis et al., 2021). Furthermore, in December 2020 in Russia, seven poultry farm workers were infected with a clade 2.3.4.4b H5N8 virus (Pyankova et al., 2021). In June 2021, 2,782 outbreaks of H5N8 were reported, causing the mortality or destruction of approximately 38 million poultry in more than 25 countries (Cui et al., 2022).^1^

In conclusion, the spread of High pathogenic AI H5N8 viruses has raised serious issues for the security and conservation of animals, poultry, and even public health (Shi and Gao, 2021). All this evidence suggests that H5N8 viruses are likely to spread worldwide; therefore, continuous surveillance and vaccination of poultry are highly recommended. In this review, we describe the emergence of sporadic infection continentally, and the impacts are briefly described.

### Intra and inter-continental transmission patterns of sporadic infection of HPAI H5N8

#### Asia and Africa

A number of emergence and re-emergence studies of HPAI H5N8 strains have been reported within & across Asia & Africa. One HPAI H5N8 virus (Dkk1203) was isolated from a poultry farm in mainland China during 2009–2010. The Dkk1203 isolate derived its HA gene from the Asian H5N1 lineage. Phylogenetic analysis of the HA gene revealed that this isolate was classified into the 2.3.4 clade. Compared to H5N5 viruses that were isolated between December 2008 and January 2009, this strain has longer branches. This strain was distantly related to Eurasian N8 genotype viruses and clustered with three H3N8 viruses with an origin in Eastern Asia. Therefore, the N5 and N8 NA genes of the Dkk1203 isolate are derived from Asian viruses; however, the exact origin is not known (Zhao et al., 2013).

In a breeding duck farm on January 16, 2014, in the Jeonbuk Province of South Korea, High-pathogenic AI clinical signs, such as reduced egg production by about 60% and slightly increased mortality rates, were discovered. Moreover, on January 17 of the same year, a farmer was also diagnosed with HPAI from breeder ducks in the Donglim Reservoir (Lee et al., 2014). Also, the Donglim Reservoir had 100 Baikal teal carcasses, all of which tested positive for the high pathogenic AI H5N8 virus (Lee et al., 2014).

A few months later, in April 2014, an outbreak of the HPAI virus with the genotype H5N8, A/chicken/Kumamoto/1–7/2014, occurred in Japan (Kanehira et al., 2015). The HA clade 2.3.4.4 membership of this virus was also made known. In particular, A/broiler duck/Korea/Buan2/2014 and A/baikal teal/Korea/Donglim3/2014, HPAI H5N8 that were isolated in Korea in January 2014, all eight genomic segments displayed substantial sequence similarity (Kanehira et al., 2015). The experimental work delineated that this isolate from Japan was lethal in chickens when a higher titer of virus was used for infection; however, the chickens were unaffected when challenged with lower viral doses (Kanehira et al., 2015).

In the same year (2014), three H5N8 viruses were reported from domestic geese in mainland China. The selected strains’ sequence analyses revealed that all H5N8 viruses were direct progeny of the K1203 (H5N8)-like viruses discovered in China in 2010 and belonged to the Asian H5N1 HA lineage of clade 2.3.4.4. The recent common clade 2.3.4.4 H5N8 reassortants, which have severely damaged the poultry sector and pose a threat to public health, were created by K1203-like viruses, according to studies (Li et al., 2014).

Eight highly pathogenic H5N8 AIVs were discovered in Japan over the winter, particularly in a location where migratory birds overwinter. These isolates were divided into three groups based on genetic analysis, demonstrating that three genetic subgroups of H5N8 HPAIs circulated in these migratory birds. These findings also suggest that the migration of these birds next winter may result in the redistribution of H5N8 HPAI globally (Ozawa et al., 2015; Isoda et al., 2020).

In 2016 in Malard County of the Tehran Province and the Meighan wetland of Arak City, Markazi Province, the HA genes indicated categorization in the 2.3.4.4b subclade. Although being identified as an H5N8 2.3.4.4b virus, the A/Goose/Iran/180/2016 virus’s cluster was split from the A/Chicken/Iran/162/2016 virus. This suggests that the entry of these viruses in Iran occurred through more than one window. The most recent HPAI-H5 outbreak in Iran happened in 2015 and was entirely caused by viruses from clade 2.3.2.1c. These findings underscore the necessity to continue proper monitoring activities in the target wild and domestic bird species for early HPAI identification and show that Iran is at high risk of the importation of HPAI H5 of the A/Goose/Guangdong/1/1996 lineage from East Asia. These activities would also allow the study of the genetic and antigenic evolution of H5 HPAI clade 2.3.4.4.viruses in the region and the world (Ghafouri et al., 2017). Furthermore, it appears that migrating wild aquatic birds carried these HPAI H5N8 strains into Iran *via* the West Asia-East African flyway (Motahhar et al., 2016).

An H5N8 influenza virus of clade 2.3.4.4 outbreak was reported in 2016 in the Republic of Tyva. The H5N8 clade 2.3.4.4 virus spread over Europe in the fall. The reports provide a clear overview of the viral strains that were discovered in the Russian Federation during the spring and fall of 2016. The strains under investigation were extremely harmful to mice, and several of their antigenic and genetic characteristics were different from an H5N8 strain that was prevalent in Russia in 2014 (Marchenko et al., 2017).

The newly emerged H5N8 influenza virus was also isolated from green-winged teal ducks. The genomes of the HPAI H5N8 viruses from Egypt were also found to be related to recently identified reassortant H5N8 viruses of clade 2.3.4.4 recovered from several Eurasian nations, according to analyses of the viruses’ genomes. The Egyptian H5N8 viruses had a number of genetic shifts that likely allowed for the spread and virulence of these viruses in mammals. Instead of human-like receptors, Egyptian H5N8 viruses prefer to bind to avian-like receptors. Likewise, amantadine and neuraminidase inhibitors had little effect on the Egyptian H5N8 viruses. It is important to continue monitoring waterfowl for avian influenza because it provides early warning of specific dangers to poultry and human health (Kandeil et al., 2017). The presence of this group and clade was also found in Qinghai Lake, China, in 2016, which resulted in the deaths of wild migratory birds (Li et al., 2017). An HPAI H5N8 virus of clade 2.3.4.4b has been detected in Egypt. PA and NP gene replacement identified the strain as A/duck/Egypt/F446/2017. The Russian 2016 HPAI H5N8 virus (A/great crested grebe/Uvs-Nuur Lake/341/2016 (H5N8)) was likely the source of Egyptian H5N8 viruses, according to Bayesian phylogeographic analysis and reassortment most likely took place prior to an incursion into Egypt (Yehia et al., 2018).

In Egypt, multiple introductions of different reassorted strains have been observed. The antigenic sites A and E of the HA gene have two new mutations. With various vaccination seeds, the HA nucleotide sequence identity ranges from 77 to 90%. To determine the main reassorted strain in Egypt, full-genome sequence analysis representing various governorates and sectors has been conducted. All viruses have been shown to be identical to the clade 2.3.4.4b reassorted strain that was discovered in Germany and other nations. Examination of these viruses revealed changes unique to Egyptian strains rather than the original virus identified in 2017 (A/duck/Egypt/F446/2017), and two strains of these viruses had the novel antiviral resistance marker V27A, which indicated amantadine resistance in the M2 protein. The findings showed that circulating H5N8 viruses were more variable than prior viruses analyzed in 2016 and 2017. An early 2017 strain served as the foundation for the main reassorted virus that circulated in 2017 and 2018. To track the development of circulating viruses, it is crucial to keep up this surveillance of AIVs (Yehia et al., 2020). The Democratic Republic of the Congo strains also belongs to the same clade, 2.3.4.4B. The emergence of this clade in central Africa threatens animal health and food security (Twabela et al., 2017).

The recovered HPAI A(H5N8) viruses in Pakistan during 2018–19 belonged to clade 2.3.4.4b and were most closely related to the Saudi Arabian A(H5N8) viruses, which were most likely introduced *via* cross-border transmission from nearby regions about 3 months before the virus was discovered in domestic poultry. It was also found that, prior to the first human A(H5N8) infection in Russian poultry workers in 2020, clade 2.3.4.4b viruses underwent rapid lineage expansion in 2017 and acquired significant amino acid mutations, including mutations correlated with increased hemagglutinin affinity to human-2,6 receptors. Our findings demonstrate the necessity of routine avian influenza surveillance in Pakistan’s live bird markets in order to keep an eye out for any potential A(H5Nx) variants that might emerge from poultry populations (Ali et al., 2021). Every year, the Indus Flyway, also known as the Green Way, transports between 0.7 and 1.2 million birds from Europe, Central Asian countries, and India to Pakistan. [International Visitors: Birds Come Flying In. http://www.wildlifeofpakistan.com/PakistanBirdClub/birdcomeflyingin.html].

A thorough investigation was conducted to track the evolution of influenza viruses in poultry during the years 2020–2022 in China. A total of 35 influenza viruses, including 30 H5N8 viruses, 3 H5N1 viruses, and 2 H5N6 viruses, were isolated from chickens, ducks, and geese. The internal genes of H5N1 and H5N6 viruses shared different genetic heterogeneity with H5N8 viruses and had been reassorted with wild bird-origin H5N1 viruses from Europe. All HP H5N8 isolates were derived from clade 2.3.4.4b. The fact that practically all H5N8 viruses in China and Korea showed just one phylogenic cluster with H5N8 viruses of wild bird origin suggests that the H5N8 viruses in China were more stable. We also discovered that the main geographic source for the transmission of these H5N8 viruses to northern and eastern China is Korea. The majority of the co-circulation of H5N8 viruses took place within China, with central China serving as a seeding population during the H5N8 epidemic. Strong statistical evidence supported viral migration from wild birds to chickens and ducks, demonstrating that during 2020–2021, 2.3.4.4b H5N8 viruses with poultry origins were borne by wild birds. Multiple gene segments were also discovered to be involved in the development of severe disease due to H5N8 HPAI viruses, in mallards birds, which explains why no viral gene was found to be solely responsible for reducing the high virulence of an H5N8 virus but the PB2, M and NP segments significantly decreased mortality Our results give new insights into the dynamics of H5 subtype influenza virus evolution and transmission among poultry following the almost one-year invasion of China by novel H5N8 viruses (Leyson et al., 2021; Ye et al., 2022). In China, the re-emergence of the High Pathogenic H5N8 virus in domestic geese was also reported (Guo et al., 2021).

The establishment of novel H5N8 strains in China is frequently linked to the migration of migratory birds *via* the East Asian-Australasian Flyway. This flyway connects Siberia to Australia and includes various stopover spots in China where wild birds gather throughout their annual migration. These locations allow diverse bird species to interact and exchange influenza viruses (Li et al., 2022).

During, May 2020 in Iraq, H5N8 was reported in poultry. Complete genome sequencing delineated that a noval H5 2.3.3.4b variant had emerged. Furthermore, the long branch lengths for all segments indicated that undetected isolate was circulating for some period and possibly in galliform poultry (Lewis et al., 2021).

After outbreaks in Iraq in July 2020, H5N8 was detected in ducks, geese, and backyard chickens of Chelyabinskaya Oblast (Chelyabinsk), in southern central Russia. During August and September 2020, a total of 11 cases were detected in the Tyumen, Omsk, and Kurgan regions of Russia (Lewis et al., 2021). Wild birds were described as the cause of the incursion.

Concurrent with the H5N8 outbreak in Russia, the outbreak of H5N8 was also confirmed in several regions of Kazakhstan, including Kostanay, Akmola, and Pavlodar (Lewis et al., 2021). AI H5N8 diagnosis was confirmed by subtype-specific quantitative RT–PCR (Nagy et al., 2021). The AI H5N8 virus from Iraq and Kazakhstan shared a lot of genetic similarities, according to genetic analyses (Lewis et al., 2021).

### Europe and the Americas

In 2014, European countries, such as Germany, the United Kingdom, the Netherlands, and Italy, reported several outbreaks of H5N8 in poultry. Two different Highly pathogenic viruses, H5N2 and H5N8, were found in the United States in December 2014 in wild birds and later in backyard birds in Washington State. This sparked concerns about potential connections with recent H5N2 outbreaks in Canada and H5N8 in Asia, which is now affecting poultry farms in Europe. The continuous spread of these Eurasian HPAI H5 viruses among wild birds has a significant impact that could arise and the ensuing consequences on American poultry and wildlife rehabilitation facilities. Tundra swans (*C. columbianus*), c ommon teal (*A. crecca*), spot-billed duck (*A. poecilorhyncha*), Eurasian wigeon (*A. penelope*) and mallard, that appeared to be in good health also tested positive for the HPAI H5N8 virus, which raises the possibility that wild birds may be contributing to the spread of this High Pathogenic H5 lineage in North America (Ip et al., 2014).

With a comprehensive review of the spatiotemporal expansion and genetic characteristics of HPAI Gs/GD H5N8 from Poland’s 2019/20 epidemic, the Highly pathogenic H5 subtype of the Gs/GD lineage repeatedly invaded Poland from 2016 to 2020, posing a major threat to poultry globally. In nine Polish provinces during 2019 and 2020, 35 outbreaks in backyard and commercial poultry holdings as well as 1 incidence in a wild bird were confirmed. The majority of the outbreaks were found in the meat of ducks and turkeys. All sequenced viruses belonged to a previously unidentified genotype of HPAI H5N8 clade 2.3.4.4b and were closely related to one another. The main methods of HPAI dissemination were found to be human activity and wild birds. A review of current risk assessment techniques is necessary in light of the HPAI virus’s unusually delayed emergence (Shin et al., 2019; Śmietanka et al., 2020).

### Asia and Europe

A new wave of H5N8 outbreaks in domestic and wild birds was observed in several European nations in October 2020, including the United Kingdom, Denmark, Ireland, Germany, and the Netherlands. In August 2020, several outbreaks of the disease were confirmed from Russia in both domestic and wild birds, and the affected regions spread to Kazakhstan in mid-September. Moreover, H5N8 epidemics in domestic and/or wild birds appeared in East Asia (Japan and South Korea) and the Middle East (Israel). A unique variant between clade 2.3.4.4b and Eurasian LPAI viruses in wild birds was described as well as two different forms of HPAI H5N8 variants, one of which only belonged to clade 2.3.4.4b. The geographical areas affected have been steadily expanding, and at least 46 nations have documented highly pathogenic H5N8. with one of the human cases being related to poultry workers during an outbreak in poultry (Pyankova et al., 2021).

An influenza A (H5N8) clade 2.3.4.4b strain was recovered from a poultry worker during an outbreak of highly pathogenic H5N8 in chickens at a poultry farm in the Astrakhan region on the Volga River in southern Russia in December 2020, according to a study of a similar nature. Nasopharyngeal swabs were collected from seven poultry workers that tested positive, and two were confirmed by RT–PCR and sequencing. The seven individuals, five of whom were female and two of whom were male, ranged in age from 29 to 60. The HA gene of all five viruses obtained from birds and one from humans shared a significant degree of genetic similarity with other clades. From 2016 to 2021, viruses with the 2.3.4.4b gene were found in wild and domestic birds in Russia. human influenza A in some cases (H5) 2.3.4.4. A potential public health hazard is infections (Pyankova et al., 2021).

H5N8 clade 2.3.4.4b outbreaks were observed in Russia, the Middle East, Central Europe, and Ukraine in 2016. In the southern part of Ukraine, close to areas where migrating waterfowl congregate in large numbers, especially mute swans (*Cygnus olor*), an outbreak of HPAI strains was documented in domestic backyard poultry between 2016 and 2017. Upon sequence analysis, it was found that 2 novel H5N8 HPAI strains were isolated from domestic backyard chickens (*Gallus gallus*) and mallard duck (*Anasplatyrhynchos*). HPAI outbreaks in Ukraine underscore the ongoing need for AIV bio-monitoring, genomic sequencing, and mapping of wild bird flyways and their contacts with domestic poultry in Eurasia (Sapachova et al., 2021).

Long-distance migratory birds can play a significant role in the global spread of avian influenza viruses, notably through nesting regions in the sub-arctic. The investigation of H5N8 viral sequences, epidemiological studies, waterfowl migration, and chicken trade all revealed that wild birds can spread the virus to poultry *via* contact with infected water or surfaces. Furthermore, the chicken trade may contribute to the virus’s spread. Clade 2.3.4.4 viral hemagglutinin was discovered to be extraordinarily promiscuous, producing reassortants with diverse subtypes and potentially boosting its ability to infect different species of birds and mammals. This promiscuity is likely to have a role in its ability to quickly adapt to various hosts and settings, potentially enhancing its pandemic potential (Lycett et al., 2009).

## H5N8 evolution

### Whole genome

Gammaviruses are characterized as low pathogenic (LP) viruses or highly pathogenic (HP) viruses based on virulence in chickens. HP viruses may emerge from LP viruses through genetic mutations in wild birds (Fouchier et al., 2005). In this context, AIV subtypes H5 and H7 are characterized as HP viruses. To date, AI viruses have 16 subtypes on the basis of the Hemagglutinin gene and 9 due to the Neuraminidase gene (Webster et al., 1992; Fouchier et al., 2005).

The entire genome of HPAI H5N8, is made up of eight single-stranded RNA segments. Each segment encodes a distinct gene that is essential for the virus’s replication and infection. Polymerase Basic Protein 2 (PB2), which is roughly 2,341 nucleotides long and encodes the PB2 protein, is one of these segments. The Polymerase Basic Protein 1 (PB1) gene is approximately 2,341 nucleotides long and codes for the PB1 protein. The Polymerase Acidic Protein (PA) gene encodes the PA protein and is approximately 2,234 nucleotides long. The Hemagglutinin (HA) gene encodes the HA protein and is approximately 1,778 nucleotides long. The Nucleoprotein (NP) gene has a length of about 1,565 nucleotides and codes for the NP protein. The Neuraminidase (NA) gene encodes the NA protein and is approximately 1,413 nucleotides long. The Matrix (M) gene encodes the M1 and M2 proteins and is approximately 1,027 nucleotides long. The Non-structural protein (NS) gene has around 890 nucleotides and encodes the NS1 and NS2 proteins. It is crucial to note that the lengths provided are approximations and may differ slightly across various H5N8 strains or isolates (Bouvier and Palese, 2008).

### Hemagglutinin gene (HA)

HA gene sequence analysis was performed, and a phylogenetic tree was constructed by comparing sequences retrieved from the GISAID platform.^2^ These HPAI H5 strains belong to different groups and lineages. Sequence analysis was performed by following H5 numbering, which uncovered the genetic diversity during evolution. The cleavage site motif of HPAI H5 includes the polybasic amino acids QGERRRKKR*GLF (Perdue et al., 1997; Siddique et al., 2012), whereas in the selected isolates reported globally during different years, maximum HPAI H5N8 evolved, and the cleavage site became LREKRRKKR*GLF. Studies have demonstrated that, although HPAI H5N8 attaches to avian-like receptors, it may also attach to human virus-like receptors in the human respiratory tract. HPAI showed more affinity for cats than dogs, which were more susceptible to HPAI. It is suggested that, due to its establishment in ducts, the transmission of HPAI H5N8 viruses may modify the genetic evolution of preexisting avian poultry strains (Kim et al., 2014).

**Figure 1:**
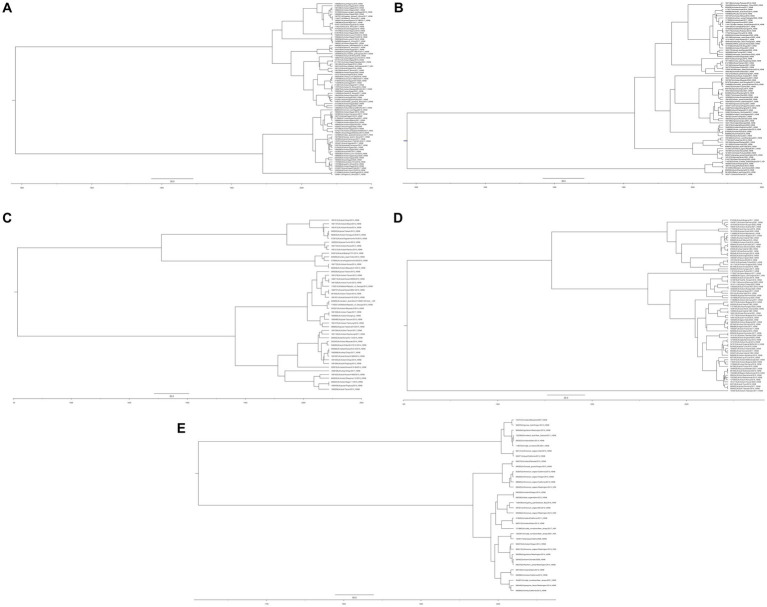
Phylogenetic analysis of the hemagglutinin gene of AIV subtype H5N8 inferred with BEAST software **(A)** Africa **(B)** Asia-group B **(C)** Asia-group C **(D)** Europe **(E)** North America and Oceania.

On the basis of similarity, H5N8 viruses evolved into three groups (Li et al., 2014). Groups I and II contain the isolates belonging to clade 2.3.4.4b and the Eurasian continent, whereas group III contains isolates from the North American lineage, with apparent divergence from those in groups I and II. Moreover, the transmission pattern of this subtype was observed in depth by reviewing the continent wide distribution in Africa (A), Asia (B & C), Europe (D), North America and Oceania (E). In this regard, HA gene sequences of selected HPAI H5N8 viruses were retrieved from the GISAID database. Initially, Bayesian evolutionary analysis was performed using BEAST version 1.10.4, and then FigTree software (v1.4.4) was used for phylogenetic tree construction, as shown (Figure 1). Moreover, No isolation has been reported from Antarctica or South America. These continent-wide sporadic infection, further clarify that the domestic birds are reassortant hosts for the emergence of novel virus subtypes and are thought to be the reservoir of AIV. The spread of these viruses could endanger the health of both humans and birds.

In addition, asparagine-linked glycosylation sites have been observed among HPAI H5 strains, revealing that some are common during evolution, whereas a number of substitutions and deletions are also seen. Siddique et al. in 2012 reported the same sites along with additional glycosylation sites at the globular head of the HA gene, which is responsible for the prediction of high efficiency of replication (Bender et al., 1999; Siddique et al., 2012). Moreover, the conserved amino acids at positions 222 glutamine and glycine at position 224 of the HA gene are responsible for avian-like receptors at the binding site that is common among all the HPAI H5 proteins selected for analysis, and similar reports are available in this context (Matrosovich et al., 1999; Smith et al., 2006; Siddique et al., 2012).

Furthermore, a number of amino acid mutations have been observed at antigenic sites, including at amino acid position 39, where glutamic acid has been shown to have mutated into glycine, S141P, K169R, D171N, A172T, R178I/R, P197S, R205N/K, and N268Y. These sites have been designated as crucial residues of the antigenic site (Kaverin et al., 2004).

In NA, PB1, PB2, PA, NP, PA, M, and NS, almost 29 molecular signatures are present that are associated with replication, virulence, transmission, and adaptation in mammals (Hiromoto et al., 2000; Shaw et al., 2001; Chen et al., 2007; Gabriel et al., 2008; Long et al., 2008; Lycett et al., 2009; Spesock et al., 2011; Hui et al., 2017; Kamal et al., 2017; Yu et al., 2017; Pulit-Penaloza et al., 2020). In this regard, a maximum of 20molecular signatures were present in HPAI H5N1/483, whereas 4–6 were present in HPAI/LPAI H5N8 viruses. The PB2 gene contains the known marker 627 K for mammalian adaptation that has only been shown to be present in 2 HPAI H5 human isolates, HPAI subtype H5N1/483 and H5N6/39715. There are a number of other mutations in the NA gene at the 96A amino acid position and the Matrix 2 gene at the S31N site that are responsible for dual resistance against antivirals, including oseltamivir and amantadine (Cheung et al., 2006; Ilyushina et al., 2010). However, some other mutations, such as R118K in the NA gene, are associated with additional resistance to zanamivir (Intharathep et al., 2008; Orozovic et al., 2011). Due to these genetic changes, adamantanes and neuraminidase inhibitors may not be able to effectively prevent the replication of these viruses in the host in this situation.

The highly pathogenic AI H5 subtype has been spreading at an unprecedented rate since 2021, which is concerning given the disease’s high mortality rate in wild birds and poultry as well as cases that have been observed in mammals and people. This could potentially lead to a future pandemic. Along with causing mass demise in a number of wild mammal species, H5 HPAI has the capacity to switch from infecting avian to mammalian hosts and develop the necessary characteristics for effective transmission from mammal to mammal. Therefore, enhanced surveillance of wild animals, large-scale animal farms, and humans handling them is urgently needed, along with improved biosecurity measures, reduction of poultry farm size and density, vaccination of poultry against HPAI, and avoidance of areas rich in water birds as a location for poultry farms. In addition, the medical sector and society need to prepare for the emergence of the human-to-human spread of H5 HPAI. It is crucial to include the community, communicate about risks, and counter intentional disinformation. The next pandemic, which could result from this AIV, should be prepared for using the lessons learned from the COVID-19 pandemic as a reference (Kuiken, 2023).

### Risk assessment and mitigation strategies

During 2020–21, in Eurasia, Europe, and Africa, emergent strains were highly pathogenic subtypes of H5N8 belonging to clade 2.3.4.4b and had a significant impact on the poultry industry. In the current scenario, an emergency has been declared for the enhancement of sero-and viro-surveillance across the globe depending on the previous outbreaks in 2005 and 2016 (Alarcon et al., 2018; Adlhoch et al., 2020). For risk mitigation strategies, an effective risk assessment needs to be performed in terms of tissue/host tropism, pathogenesis, and disease transmission and dissemination. Influenza A virus poses a continuous threat to poultry and the public due to its evolutionary mechanism through reassortment.

HPAI are extremely risky to poultry if not properly vaccinated. The low pathogenic H7N9 virus which emerged in 2013, was converted into high pathogenic due to mutations in early 2017, caused the death of millions of chickens to control the outbreak (Shi et al., 2017; Zeng et al., 2018). The use of H7N9 vaccines effectively controlled the circulation of this virus both in poultry and humans (Zeng et al., 2020). Since 2004 vaccines are in in use against H5 avian viruses in China (Zeng et al., 2020). Since the emergence of H7N9 in 2017, a bivalent inactivated vaccine against H5/H7 was developed to control both H5 and H7 in poultry in China (Shi et al., 2018; Zeng et al., 2018). Currently, a trivalent vaccine-H5/H7 which contain Re-11, Re-12 and H7-Re3 vaccine seed viruses are in use. This trivalent vaccine was generated by reverse genetics, and HA genes were derived from A/duck/Guizhou/S4184/2017(H5N6) (DK/GZ/S4184/17) (a clade 2.3.4.4 h virus), A/chicken/Liaoning/SD007/2017 (H5N1) (CK/LN/SD007/17) (a clade 2.3.2.1d virus), and A/chicken/Inner Mongolia/SD010/2019 (H7N9) (CK/IM/SD010/2019), respectively (Zeng et al., 2020; Cui et al., 2022). Although the newly emerged H5N8 viruses differ antigenically from currently used vaccines, poultry birds vaccinated in routine with current vaccines still completely protect against H5N8 virus challenge (Cui et al., 2022). In another recent study (Niqueux et al., 2023), the efficacy of three vaccines was determined against the HPAI A/decoy duck/France/161105a/2016 (H5N8), clade 2.3.3.4b. The first vaccine (Vac1), was derived from HA gene clade 2.3.4.4b A (H5N8) HPAI, the second vaccine (Vac2) used was a commercial bivalent adjuvanted vaccine that contained an expressed HA modified from clade 2.3.2 A (H5N1) HPAI. The third vaccine (Vac3) also incorporated a homologous 2.3.4.4b H5 HA gene. Vac2 partly decreased the respiratory and intestinal excretion of challenge virus, Vac3 completely abolished cloacal shedding while Vac1 abolished oropharyngeal and cloacal shedding to almost undetectable levels. These results provided significant insights in the immunogenicity of recombinant H5 vaccines in mule ducks (Niqueux et al., 2023). Since the H5N8 viruses have been detected in a wide range of wild birds across the globe, therefore it could spread worldwide and can be very lethal to poultry. Therefore, homologous vaccination practices need to be introduced for the control and transmission of the disease, as the exact information on the disease and transmission is still not clear. The Iraqi-like strains are dispersed through poultry or indirect transmission in central Asia. In 2014–2017, there was little evidence of reassortment of HPAI H5N8 and H5N1 viruses in wild birds, as dispersal was unclear, but later, evidence of reassortment was found to be substantive, whereas in Europe in 2020, the emerging HPAI H5N8 strain was clearly a combination of sub-Saharan African viruses with a Eurasian LPAIV origin. Despite the implementation of biosecurity measures, several outbreaks of HPAI H5N8 strains were reported in France during 2016–2017, possibly due to airborne viral transmission. The area around the poultry facilities, almost 50–110 m, is considered contaminated with varied viral concentrations (Scoizec et al., 2018). In case of outbreaks, depopulation methods need to be wisely implemented to further control the air-borne contamination of influenza viruses, which could result in instant mass culling.

## Conclusions and future perspectives

This study backs up the hypothesis that asymptomatic migrating birds may have assisted viral development and reassortment as well as regional transmission of HPAI subtype H5N8. Another evidence that rapid and active mutation and reassortment of H5 subtypes may occur in these hosts comes from the HPAI subtypes H5N1 and H5N8 coinfecting and cocirculating in migratory ducks. Therefore, intersectoral alliance and coaction for mitigating avian influenza outbreaks based on the One Health approach that is worthwhile and advisable. This review discusses knowledge of the disease’s nature, distribution, epidemiology, applied surveillance techniques, diagnosis, and control approaches as they related to Sahelian Africa and its surrounding suburbs. Understanding of the influenza virus and its footprint on the well-being of humans and animals would aid in better preparing for the erratic/capricious challenges posed by this infectious disease.

Continuous vigilance, strengthening biosecurity, and intensifying surveillance in wild birds are needed to better manage the risk of HPAI occurrence in the future. Moreover, high-risk countries should vaccinate their poultry birds to prevent further outbreaks of HPAI H5N8. This review clearly indicates that HPAI H5N8 is a threat from a poultry standpoint and public perspective and that continuous surveillance and further epidemiological studies are needed.

## Author contributions

ZX: conceptualization, supervision, and funding acquisition. SR: wrote the manuscript. FR, SM, and AA: edited and proof read the manuscript. ML, SL, and LX: collected data and revised the manuscript. All authors contributed to the article and approved the submitted version.

## Funding

This project was and funded by grants from Guangxi Science and Technology Project (no. AB21076004), Guangxi BaGui Scholars Program Foundation (2019A50).

## Conflict of interest

SR and SM were employed by Sadiq Poultry Pvt. Ltd.

The remaining authors declare that the research was conducted in the absence of any commercial or financial relationships that could be construed as a potential conflict of interest.

## Publisher’s note

All claims expressed in this article are solely those of the authors and do not necessarily represent those of their affiliated organizations, or those of the publisher, the editors and the reviewers. Any product that may be evaluated in this article, or claim that may be made by its manufacturer, is not guaranteed or endorsed by the publisher.

## Footnotes

^1^ http://empres-i.fao.org/eipws3g/

^2^ http://platform.gisaid.org/
